# Validation of the Finnish Diabetes Risk Score (FINDRISC) in a Central‐European Population for the Prediction of Cumulative Incidence of Type 2 Diabetes Over 8‐Years—Follow‐Up of the Budakalász Health Examination Survey (BHES)

**DOI:** 10.1002/dmrr.70105

**Published:** 2025-11-12

**Authors:** Zsolt Bagyura, Loretta Zsuzsa Kiss, István Panykó, Beatrix Annamária Domján, Pál Soós, Zsolt Szelid, Béla Merkely, Ádám Gyula Tabák

**Affiliations:** ^1^ Heart and Vascular Center Semmelweis University Budapest Hungary; ^2^ Department of Internal Medicine and Oncology Semmelweis University Faculty of Medicine Budapest Hungary; ^3^ Károly Rácz Conservative Medicine Division Doctoral College Semmelweis University Budapest Hungary; ^4^ Department of Gynaecology and Obstetrics Komárom‐Esztergom County Szent Borbála Hospital Tatabánya Hungary; ^5^ Institute of Preventive Medicine and Public Health Semmelweis University Faculty of Medicine Budapest Hungary; ^6^ UCL Brain Sciences University College London London UK

**Keywords:** FINDRISC—Finnish diabetes risk score, recalibration, T2DM—type 2 diabetes mellitus, validation

## Abstract

**Aims:**

FINDRISC is widely used to assess 10‐year incidence of drug‐treated type 2 diabetes; however, it may require recalibration before implementation in new populations. Thus, we investigated the performance of FINDRISC and recalibrated it in the Hungarian population.

**Methods:**

8‐year follow‐up data for incident type 2 diabetes was ascertained from the reimbursement database of Hungary for 2059 diabetes‐free participants of a voluntary survey (2011–2013). Incident diabetes was based on repeated prescription of antidiabetic medications. Discrimination of the original and the recalibrated (multiple logistic regression) FINDRISC was compared using ROC analysis.

**Results:**

279 (13.6%) incident diabetes cases were found. Age, waist circumference, antihypertensive treatment, and history of elevated blood glucose were independent predictors of incident diabetes. Re‐estimating the weights improved discrimination ([AUC]: 0.68 [95% CI 0.65–0.71] vs. original: 0.66 [95% CI 0.63–0.69], *p* = 0.02). Even after the omission of variables non‐independent predictors of diabetes, the AUC remained better than the original score and similar to the reweighted score (AUC: 0.68 [95% CI 0.65–0.71] vs. original *p* = 0.04 vs. reweighted model *p* = 0.83). Discrimination was worse for those ≥ 65 years versus younger people.

**Conclusions:**

Validation and recalibration are important steps before using the FINDRISC in a population different from the derivation cohort. Omission of some variables (physical activity, fruit and vegetable consumption, and family history of diabetes) that are not readily available did not significantly worsen the performance of the model. FINDRISC may not be a good predictor of incident diabetes in older populations.

**Abbreviations**
ATC: Anatomical Therapeutic Chemical code
AUC: area under the curve
BHES: Budakalász Health Examination Survey
FINDRISC: Finnish Diabetes Risk Score
HbA1c: glycated haemoglobin A1c
NEAK: National Health Insurance Fund of Hungary
OGTT: oral glucose tolerance test
ROC: receiver operating characteristic
T1DM: type 1 diabetes mellitus
T2DM: type 2 diabetes mellitus

## 1 Introduction

The worldwide prevalence of type 2 diabetes mellitus (T2DM) is increasing unabated [1]. Approximately 537 million persons were affected in 2021, and projections predict a 46% increase by 2045 [2]. Despite novel diagnostic methods, a notable proportion of diabetes cases remain undiagnosed [3].

Furthermore, there is ample evidence that early interventions, including lifestyle changes and medications, are capable of preventing or delaying the manifestation of diabetes [4]. Therefore, the detection of people at increased risk is of great importance. However, standard diagnostic tests, such as glycated haemoglobin A1c (HbA1c) or the oral glucose tolerance test (OGTT), are expensive and time consuming and hinder their use in the general population [5].

To overcome this problem and optimise the use of glycaemic tests, several prediction tools were developed [6]. The Finnish Diabetes Risk Score (FINDRISC) was developed in a Finish population and estimates 10‐year risk of incident diabetes. FINDRISC is a simple, questionnaire‐based tool that flags high‐risk individuals who may benefit most from interventions. Since its development, the score has been validated and is widely applied in different European populations [7], and in different ethnicities [8, 9, 10] by reweighting and recalibration.

Currently, it is unknown how the original score performs in a Central‐European population. Given this uncertainty, we aimed to evaluate the performance of the original questionnaire and to optimise it by reweighting and recalibration using follow‐up data from a population‐based health survey from Hungary [11].

## 2 Methods

Our prospective cohort study is based on data from a voluntary health examination survey (Budakalász Health Examination Survey—BHES) [12] performed in 2011–2013 in Budakalász, a town with 10,619 (in 2011) inhabitants in the agglomeration of Budapest. Adult volunteer town dwellers were invited to visit the screening centre.

The survey included 3 parts: An interview was conducted based on the European Health Interview survey questionnaire collecting data on health status (self‐perceived health, chronic diseases) and health determinants (smoking, alcohol consumption, physical activity, dietary habits).

The medical examination included medical history, symptoms of cardiovascular diseases, family history, current medications, resting ECG, anthropometrics, transthoracic echocardiography, and carotid ultrasound scan.

Non‐fasting laboratory tests included blood cell counts, liver and kidney function, blood lipids and high sensitivity C‐reactive protein.

Given that Hungary has a socialised healthcare system, an almost complete follow‐up was possible by using databases of the National Health Insurance Fund of Hungary (NEAK). All participants of BHES were flagged using their unique social security number in the prescription registry and were followed up for incident diabetes for 8 years after the baseline examination.

### 2.1 Participants

Of the 8281 eligible adults (≥ 20 years), 2429 (29.3%) participated. After exclusions made by design or due to missing data, the final analytical sample included 2059 people (96.9%) (Figure S1).

Prevalence of diabetes at baseline was based on self‐reports and medical history taken by a physician using self‐reports, current medication lists, and available discharge and outpatient documents.

### 2.2 Outcome

The outcome is the cumulative incidence of drug‐treated diabetes from baseline (18/APR/2011‐16/DEC/2013) over an 8‐year follow‐up for everyone. Prescription data were extracted from the database of the NEAK for the period 18/APR/2011 to 16/DEC/2021. Given that all antidiabetic medications require a prescription, all drug‐treated diabetes cases should be recorded. Incident drug‐treated diabetes was defined as at least 2 redeemed prescriptions for any reimbursed antidiabetic medication (Anatomical Therapeutic Chemical Classification System code A10*) over follow‐up. The date of diagnosis was the date of the first filled prescription.

Given that insulin treated diabetes is frequently coded as type 1 diabetes (T1DM) in Hungary and the proportion among adult‐onset diabetes cases was 6%–7% according to data from the US and Sweden, we decided not to use the ICD codes to determine the type of diabetes and included all incident diabetes cases in our analysis. [13, 14] We think that the misclassification of the outcome related to metformin use in non‐diabetic people is unlikely as metformin use for prediabetes or polycystic ovarian syndrome is considered as off label use and is not reimbursed by NEAK. Furthermore, all participants who reported metformin use at baseline had a diabetes diagnosis. Similarly, liraglutide may have been given to people free of diabetes for obesity. [15] However, liraglutide is not reimbursed for obesity and thus, it is rarely used in Hungary. [16].

### 2.3 FINDRISC

Participants' *sex* and *age* at baseline were derived from the health questionnaire.

Data on *daily fruit/vegetable consumption* was derived using the following question: ‘How frequently do you eat fruits or vegetables?’. People that responded either at least twice daily or daily were coded as positive.

*Height and weight* were measured according to standardised protocols in light clothing without shoes. Height was measured to the nearest centimetre using a stadiometer with the Frankfort plane in the horizontal position and weight to the nearest 0.1 kg on a calibrated electronic scale. *Body mass index* (BMI) was calculated as weight (kg)/height (cm^2^) [2].

*Waist circumference* was measured to the nearest centimetre mid‐way between the iliac crest and lower rib using a measuring tape.

*Physical activity* was defined as positive if the participant answered the following question positively: ‘Do you usually have at least 30 min of physical activity at work or during leisure time?’.

*Treated hypertension* was defined as medically treated hypertension in the history. Similarly, the examination included a specific question on an *increased blood glucose value in the medical history (yes/no)*.

*Family history of diabetes* was collected separately and then coded as first‐degree relatives (mother, father, brothers, sisters, own children) and other relatives.

Using the non‐fasting sample, HbA1c level was measured using haemolysed whole blood by a turbidimetric inhibition immunoassay method (Tina‐quant Haemoglobin A1c Gen.2, Roche Diagnostics, Germany) in the Department of Laboratory Medicine, Semmelweis University.

For each participant, FINDRISC was calculated according to the weighting published by Lindström et al. [17] (Supporting Information S1: Appendix) High risk of diabetes for the current analysis was defined as a FINDRISC of ≥ 12.

### 2.4 Statistical Analysis

We provide descriptive statistics for baseline characteristics of participants by incident diabetes status.

Similar to the original derivation of FINDRISC, our prediction model is based on logistic regression. However, our follow‐up was shorter (8 vs. 10 years) than that for the FINDRISC derivation cohort; thus, the main outcome of our analysis was the 8‐year cumulative incidence of T2DM.

For the analysis, we followed the TRIPOD + AI recommendation [18]. We developed 3 prediction models: First, FINDRISC was calculated using the original point system and entered into a model as the sole predictor (*original model*). Then, we entered all as independent predictors (*reweighted model*). Finally, we used a stepwise elimination procedure to remove non‐significant predictors to reach the most parsimonious model (*optimised model*) for diabetes prediction.

The beta coefficients for each model were translated to points using 2 different methods: The first replicated the one used for the FINDRISC derivation (Supporting Information S1) [19]. The second replicates the scoring used to derive the Framingham risk scores [20]. In the present study, the actual score was derived as score = round (beta/0.2). The advantages of the latter method are that (1) it allows the derivation of negative points in cases where the risk in a given group is smaller than in the reference group and (2) the points have a direct linear relationship with the actual beta values.

*Model fit* was characterised by log likelihood (LL) function and Akaike information criteria (AIC) [21]. *Discrimination* was characterised by the area under the receiver‐operator characteristic (ROC) curves (AUC) with their respective 95% confidence intervals (CI). The ROCs of the different models were compared using ROC analysis in SPSS with the paired‐sample design. In line with the TRIPOD + AI recommendation, we tested the fairness of the model through a post hoc ROC analysis stratified by age (≤ vs. > 65 years) [18]. This analysis is especially important as the original derivation cohort of the FINDRISC was limited to persons < 65 years of age; however, the prediction model is widely used in older people [17, 19]. *Calibration* was investigated by the Stata package bsvalidation [22] that provides apparent and optimism adjusted performance measures based on bootstrapping: AUC, expected to observed (E/O) ratio, calibration in the large (CITL), and calibration slope [23]. Finally, *net reclassification improvement* (NRI) with 95% CIs was calculated for the reweighted and the optimised models compared to the original using 12 points as the cutoff for the original FINDRISC and the Youden index (the cutoff where the sum of sensitivity and specificity is maximal) for the reweighted and optimised scores.

All analyses were performed using SPSS 27.0 (SPSS, Chicago, IL, USA) and StataNow 18.5 (StataCorp LLC, TX, USA). Statistical significance was set at a two‐sided *p* value < 0.05.

## 3 Results

### 3.1 Baseline Characteristics by Incident Diabetes Status

Over 8 years of follow‐up, altogether 279 participants (13.6%) developed diabetes. Incident diabetes cases were 4.4 years older, 1.7 cm shorter, 4.8 kg heavier, had a 2.2 kg/m2 higher BMI, 5.6 cm larger waist, 0.4% higher HbA1c, and a 2.5‐point higher FINDRISC at baseline compared to controls. Furthermore, they were less frequently daily fruit/vegetable consumers (OR 0.71), had more frequently treated hypertension (OR 2.00) and elevated blood glucose value in their history (OR 3.23) (Table 1).

**TABLE 1:** Baseline characteristics by incident diabetes mellitus status.

	No diabetes	Incident diabetes
*n*	1780	279

	Mean ± SD/*n* (%)	Mean ± SD/*n* (%)
Age (years)	53.17 ± 14.80	57.56 ± 13.05
Male, *n* (%)	741 (41.63%)	106 (37.99%)
Height (cm)	168.1 ± 10.12	166.5 ± 9.71
Weight (kg)	77.4 ± 16.14	82.2 ± 18.57
BMI (kg/m^2^)	27.3 ± 4.88	29.6 ± 5.60
Waist circumference (cm)	95.9 ± 12.72	101.5 ± 13.38
HbA1c (%)	5.54 ± 0.36	6.03 ± 0.89
Treated hypertension, *n* (%)	604 (33.93%)	142 (50.90%)
History of high blood glucose, *n* (%)	85 (4.78%)	39 (13.98%)
≥ Daily consumption of fruit and vegetable, *n* (%)	1323 (74.33%)	224 (80.29%)
Physical activity ≥ 30 min/day, *n* (%)	1145 (64.33%)	177 (63.44%)
Family history of diabetes, *n* (%)		
Second degree relative	107 (6.01%)	11 (3.94%)
First degree relative	254 (14.27%)	52 (18.64%)
None	1419 (79.72%)	216 (12.13%)
FINDRISC score	9.48 ± 4.47	12.01 ± 4.37

Abbreviations: BMI, body mass index; DM, diabetes mellitus; FINDRISC, Finnish diabetes Risk score.

### 3.2 Prediction Model Performance

Compared to the original FINDRISC, the reweighted score had substantially different weights based on the logistic regression model. The effect of age was stronger based on the model betas in the original cohort compared with the reweighted model drawn in BHES. We found no increase in the risk of diabetes among those 45–55 years of age in BHES (*p* for difference< 0.05) and the association remained shallower in BHES compared to the original cohort. Similarly, the role of BMI seemed to be weaker in BHES compared with the original cohort, especially in those with a BMI > 30 kg/m2 (*p* for difference = 0.06). We observed similar trends for waist circumference. We also observed a weaker association between the use of anti‐hypertensive medications as well as a history of high blood glucose and diabetes risk in BHES compared to the original cohort (both *p* for difference < 0.05), while the role of fruit/vegetable consumption was non‐significant in both studies without a significant difference (Table 2).

**TABLE 2:** Multiple logistic regression models predicting incident type 2 diabetes mellitus in the original FINDRISC derivation cohort and the Budakalász Health Examination Survey.

	Original FINDRISC model			Reweighted FINDRISC model				Optimised FINDRISC model				*p* value^a^
	OR (95% CI)	FINDRISC	Framingham	OR (95% CI)	*p*	FINDRISC	Framingham	OR (95% CI)	*p*	FINDRISC	Framingham	Original versus. reweighted FINDRISC score
Age (years)												
< 45	1 (ref)	0	0	1 (ref)		0	0	1 (ref)		0	0	
45–54	1.92 (1.13–3.25)	2	3	0.96 (0.61–1.53)	0.87	0	0	0.99 (0.62–1.56)	0.96	0	0	0.03
55–64	2.56 (1.53–4.28)	3	5	1.54 (1.03–2.30)	0.04	2	2	1.57 (1.05–2.34)	0.03	2	2	0.06
> = 64	NA	4		1.34 (0.88–2.04)	0.17	2	1	1.36 (0.90–2.04)	0.15	2	2	
Body mass index (kg/m^2^)												
< 25	1 (ref)	0		1 (ref)		0	0	1 (ref)		0	0	
25–29.99	1.02 (0.48–2.15)	1	0	0.77 (0.52–1.14)	0.19	0	−1	0.77 (0.51–1.15)	0.20	0	−1	0.26
> = 30	2.55 (1.10–5.92)	3	4	1.20 (0.78–1.85)	0.40	1	1	1.20 (0.77–1.88)	0.42	1	1	0.06
Waist circumference (cm)												
Female < 80; male < 94	1 (ref)	0	0	1 (ref)		0	0	1 (ref)		0	0	
Female 80–87.99; male 94–101.99	2.78 (1.43–5.40)	3	5	1.66 (0.96–2.86)	0.07	2	3	1.67 (0.96–2.88)	0.07	2	3	0.12
Female > = 88; male > = 102	4.16 (2.00–8.63)	4	7	2.33 (1.33–4.08)	0.	3	4	2.37 (1.33–4.23)	0.	3	4	0.11
≥ Daily consumption of fruit and vegetable												
Yes	1 (ref)	0	0	1 (ref)		0	0					
No	1.18 (0.85–1.64)	1	1	1.30 (0.94–1.79)	0.11	2	1					0.34
Treated hypertension												
No	1 (ref)	0	0	1 (ref)		0	0	1 (ref)		0	0	
Yes	2.04 (1.45–2.88)	2	4	1.36 (1.01–1.83)	0.04	2	2	1.35 (1.00–1.82)	0.05	2	2	0.04
History of high blood glucose												
No	1 (ref)	0	0	1 (ref)		0	0	1 (ref)		0	0	
Yes	9.61 (6.31–14.63)	5	11	2.86 (1.87–4.36)	0.	3	5	2.96 (1.95–4.50)	0.	3	4	0.
Physical activity ≥ 30 min/day, *n* (%)												
Yes	1 (ref)	0	0	1 (ref)		0	0					
No	1.31 (0.88–1.95)	2	1	0.94 (0.71–1.23)	0.64	0	0					0.09
Family history of diabetes												
None	1 (ref)	0	0	1 (ref)			0					
Second degree relative	NA	3		0.89 (0.46–1.73)	0.73	0	−1					
First degree relative	NA	5		1.25 (0.88–1.77)	0.21	2	1					

*Note:* Odds ratios (OR) and 95% confidence intervals (95% CI) for the original FINDRISC are derived from Lindstrom et al. [19]. FINDRISC: Score points estimated based on the β‐coefficients of the logistic model according to Lindstrom et al. [19]. Framingham: Score points estimated based on the β‐coefficients of the logistic model according to Sullivan et al. [20].

Abbreviations: BHES, Budakalász health examination survey; FINDRISC, Finnish diabetes risk score; T2DM type 2 diabetes mellitus.

^a^ Data show the *p* for the difference in odds ratios between this study (Reweighted FINDRISC model) and the original FINDRISC model [19].

When we used stepwise elimination of non‐significant variables from the reweighted model, overall performance of the final model remained similar to that of the reweighted model (LL ratio test *p* = 0.33) with the use of age, BMI, waist circumference, use of anti‐hypertensive medications, and history of high blood glucose remaining significant predictors. The weight of each individual predictor remained very similar to that in the reweighted model, reflected by the fact that scores based on either method of calculation remained almost unchanged (Table 2).

### 3.3 Model Discrimination

**FIGURE 1:**
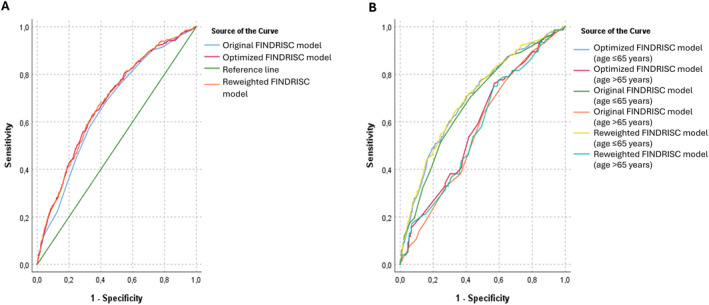
Receiver operating characteristic (ROC) curves of the different models for the prediction of cumulative incidence of type 2 diabetes mellitus overall (panel A) and stratified by age (≤ 65 vs. > 65 years; panel B) during 8 years of follow‐up of the Budakalász Health Survey. FINDRISC: Finnish Diabetes Risk score.

Overall discrimination was poor to fair based on the CIs of the AUC values for all models. (Figure 1A)

Using the optimal cutoff (based on the Youden index) gave slightly higher sensitivities (original: 68.5% vs. reweighted: 66.7% vs. optimised: 62.7%) but lower specificities (56.9% vs. 61.7% vs. 65.5%, respectively) for the original score with positive predictive values (PPV) in the range of 15%–16%, and negative predictive value (NPV) of 92% (Table 3).

**TABLE 3:** Sensitivity, specificity, as well as positive and negative predictive values of the original, reweighted and optimised FINDRISC scores for the prediction of cumulative incidence of type 2 diabetes mellitus during 8 years of follow‐up of the Budakalász Health Survey.

	Original FINDRISC score		Reweighted FINDRISC score		Optimised FINDRISC score	
	Estimate	95% CI	Estimate	95% CI	Estimate	95% CI
Cut‐off score	> = 12		> = 9		> = 9	
Sensitivity (%)	68.5	66.5–70.5	66.7	64.6–68.7	62.7	60.6–64.8
Specificity (%)	56.9	54.7–59.0	61.7	59.6–63.8	65.5	63.5–67.6
Positive predictive value (%)	15.4	13.9–17.0	16.2	14.6–17.8	16.4	14.8–18.0
Negative predictive value (%)	92	90.8–93.2	92.2	91.0–93.4	91.8	90.6–93.0

*Note:* Cut‐off values are based on the Youden index.

Abbreviations: NPV, negative predictive value; PPV, positive predictive value.

**FIGURE 2:**
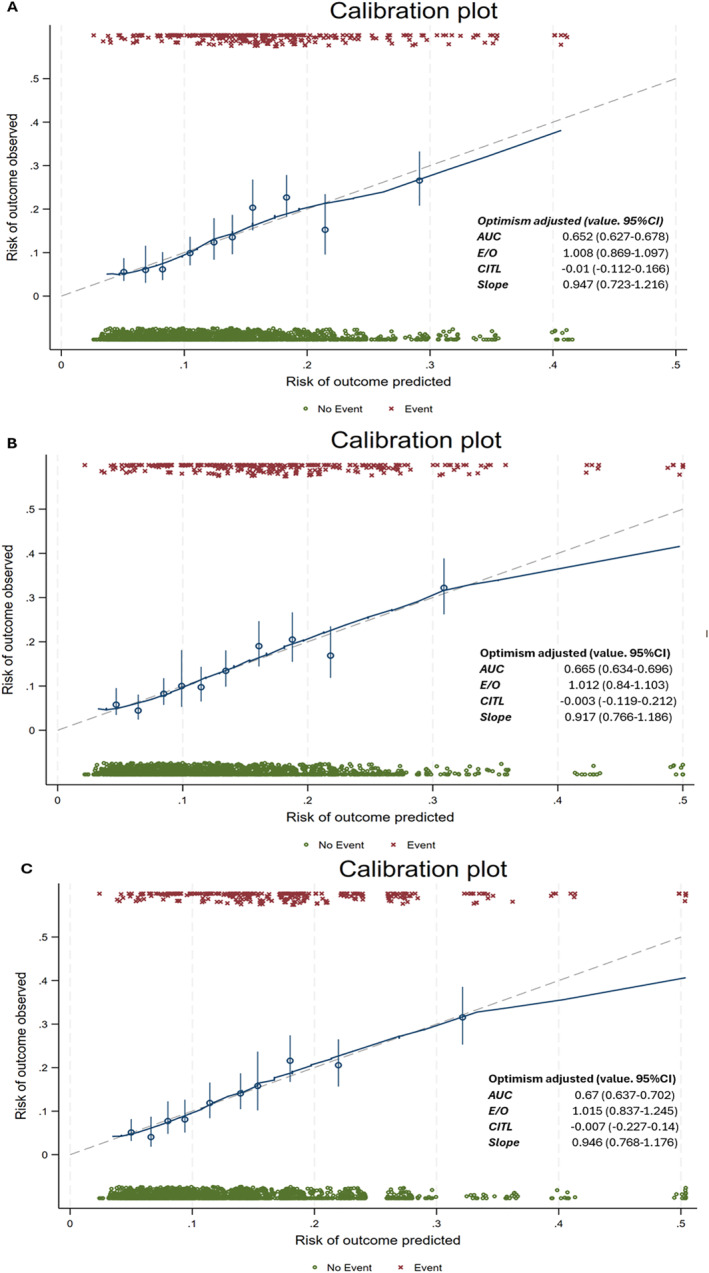
Predicted and observed incident cases of type 2 diabetes mellitus (T2DM) by deciles of the original (panel A), reweighted (panel B) and optimised (panel C) FINDRISC scores during 8 years of follow‐up of the Budakalász Health Survey. 95% CI: 95% confidence interval, AUC: area under the receiver‐operator characteristics curve, CITL: calibration‐in‐the‐large, E/O: Expected to observed ratio, FINDRISC score: Finnish Diabetes Risk score, Slope: calibration slope, T2DM: type 2 diabetes mellitus.

Both the reweighted and the optimised scores had slightly but significantly higher AUC compared to the original FINDRISC (*p* reweighted vs. original = 0.015, *p* optimised vs. original = 0.035, *p* reweighted vs. optimised = 0.83). Adjustment for optimism using bootstrapping slightly modified model discrimination (Figure 1A, Figure 2).

### 3.4 Post‐Hoc ROC Analysis Stratified by Age

Apparent discrimination was significantly worse in older participants with > 0.10 larger AUCs for younger versus. older people (*p* < 0.005 for all comparisons) (Figure 1B).

### 3.5 Model Calibration

Both the apparent and optimism adjusted calibration statistics as well as calibration plots suggested good calibration arguing against overfitting being an issue (Figure 2).

### 3.6 Model Reclassification

We calculated NRIs for the reweighted and the optimised scores compared to the original FINDRISC using 12 as the cutoff value for the original score and the values with the largest Youden index for the reweighted and the optimised scores. We found non‐significantly improved NRIs with the reweighted (NRI: 2.3%, SE: 2.3%, *p* = 0.35) and the optimised scores (NRI: 3.3%, SE 2.7%, *p* = 0.22).

## 4 Discussion

Our study is the first to externally validate the FINDRISC in a Hungarian population. Using 8‐year follow‐up data of the BHES based on linkage to the prescription database of the NEAK, the cumulative incidence of treated diabetes was 13.6%. We used logistic regression to reweight the scores and then stepwise elimination of non‐significant risk factors (fruit/vegetable consumption, daily physical activity, and family history of diabetes) to reach the most parsimonious model. The weights associated with individual components of the FINDRISC were substantially different from the original weights, suggesting that the characteristics of the derivation cohort are important determinants of these weights. Model performance and discrimination improved slightly but significantly after reweighting and optimisation; however, discrimination remained fair to poor with AUCs between 0.65 and 0.7. Model calibration suggested no overfitting. Reclassification improved non‐significantly with the reweighting and optimisation.

### 4.1 Practical Use of Risk Prediction for Diabetes Prevention

As highlighted before, accurate predictive models are of utmost importance [4, 24, 25]. Identification of prediabetes requires invasive testing and thus its use for population level screening may not be feasible. This notion led to the development of several risk scores using factors readily available or non‐invasively collectable clinical data. In general, these prediction models have fair discrimination in their derivation cohorts with AUC values of 0.7–0.8 [4, 26].

In clinical practice, a two‐stage process could be utilised. Diabetes prediction models allow preliminary risk assessment with little effort and cost. Laboratory measures, especially glycaemic values, can significantly improve the results of non‐invasive models with improvements in AUC values of 0.05–0.1 [26] It should be noted that some of these models (such as the FINDRISC) were originally developed to predict the risk of undiagnosed diabetes and were then tested for incident disease. Usually, these models have better discrimination for undiagnosed versus. incident diabetes [4, 26].

### 4.2 Results in Context of the Literature

#### 4.2.1 Discrimination

Although there is no overall agreement on the best prediction algorithm for incident diabetes in the literature, FINDRISC is widely accepted and has been widely validated [4, 6, 26]. Most of these studies reported somewhat better discrimination (AUC values of 0.71–0.85) compared to our study (0.66) [19, 27, 28, 29, 30, 31, 32, 33, 34, 35]. The only population‐based study with a similarly poor discrimination is the Botnia study [36]. It is striking that the studies with the higher AUC values either have an upper age cutoff of 65 [19, 29, 33], or included only very few or no people aged over 75 [7, 27, 30, 31, 32, 35]. In contrast, both our study and the Botnia study had no upper age limit and included a substantial number of very old people [36]. This finding may suggest that the inclusion of very old people (those surviving the life expectancy of a given population) may worsen predictive power, probably through the healthy survival effect. This is further supported by the fact that the prevalence of diabetes (and consequently its incidence) decreases after the age of 70 in the Hungarian population [3, 37]. Indeed, our results of a post hoc analysis stratified by age confirm worse discrimination in those 65 years or older. This finding is especially important as the FINDRISC model was extended to older people (> 65 years of age) with the expectation that it performs similarly in younger and older populations without the formal evaluation of fairness strongly emphasised by the TRIPOD + AI statement [17, 18].

#### 4.2.2 Cutoff Value

Most reports, like us, used a cutoff of 12 as a marker of high risk. This value in most studies (including ours) is close to the Youden index and ranges from 9 to 12. Most studies report similar sensitivities to ours in the range of 63%–70% except for the derivation cohort, where the sensitivity is 77%. In contrast, our study has lower specificity (57%) compared to the other studies (65%–85%) leading to the overall worse discrimination [19, 27, 30, 34, 35].

Our study has a higher PPV and lower NPV compared to most studies, which means that a value below the cutoff cannot be used to safely rule out diabetes development [7, 19, 30].

#### 4.2.3 Reweighting the Original FINDRISC

There is limited data on reweighting the original FINDRISC. Reweighting slightly but significantly improved model prediction by 2% both in our study and in the multi‐country cohort of Alssema et al. [28] Although this overall moderate improvement does not necessarily support reweighting of the prediction model when used in populations similar to the derivation cohort, the relative weight of the individual risk factors shows substantial and significant changes. While the odds ratios associated with the different age groups were similar in the original cohort and in DETECT‐2, our study showed a much weaker age dependence. This may relate to the healthy survival effect detailed before. Similarly, the associations with BMI, waist circumference, and the use of antihypertensives were much weaker in our study, probably related to the fact that most elderly people are on antihypertensives but are at low risk of diabetes. The role of the history of elevated blood glucose cannot be compared because of the different definitions used in the 3 studies. A family history of diabetes was not reported in the original cohort but was a strong risk factor in DETECT‐2, while it was insignificant in our population. Physical activity and fruit/vegetable consumption were not significant factors in the original or in our study. It should also be noted that the confidence intervals of all coefficients in our study overlapped with those in the individual studies in DETECT‐2 [7, 19].

These results suggest that while the weight of the individual risk factors shows a wide range, the overall prediction remains transferable to different populations. This notion is further supported by the fact that although reweighting the Framingham hypertension risk score improved its prediction in the Whitehall II study, reweighting led to only clinically non‐significant reclassification improvement [38].

#### 4.2.4 Calibration

We found no major miscalibration in our study similar to DETECT‐2 [7]. It seems that the calibration of risk scores could remain stable if the derivation of the risk score is adequate [38].

#### 4.2.5 Model Optimisation

Some of the risk factors (such as diet, physical activity) included in the FINDRISC were not associated with significantly increased risk but were retained because they are important components of the pathophysiology of T2DM. Similarly, we found that these factors were not improving model fit, and that their omission from the risk score did not worsen discrimination. Li et al. and Salinero‐Fort et al. both reported that with the use of a limited number of risk factors, unknown diabetes can be detected with similar power than with the use of the full FINDRISC [31, 39]. While a simplified risk score could substantially improve the usability of a diabetes prediction model, our findings require replication in other prospective studies.

### 4.3 Strengths and Limitations

Our study benefits from its relatively large size that provides adequate statistical power for model development. The relatively long follow‐up time also improves statistical power, as the cumulative incidence of diabetes is almost 15%. Most predictors and the outcome are defined in the same way as in the original derivation study, thus providing reassurance of the validity of our findings. The fact that FINDRISC is implemented in Hungary for the screening of high‐risk populations and advocated by the Hungarian Diabetes Association with the same cutoff value used in our study gives special importance to our analysis. While most studies on risk prediction only report model fit and discrimination, we included other metrics of performance such as calibration and reclassification.

Our study has certain limitations that must be acknowledged. While the sample size was relatively large, the response rate was low, potentially leading to selection bias based on availability and health characteristics. Furthermore, the role of misclassification of the outcome cannot be excluded. First, approximately 5%–7% of our incident cases could have T1DM that could probably worsen the performance of our prediction models, as T1DM cases have rarely shown risk factors of T2DM. Second, metformin use in non‐diabetes cases cannot be excluded, although none of the metformin users were free from diabetes at baseline. Although we have the exact date of diabetes diagnosis (first prescription), we used logistic regression instead of a time‐to‐event analysis. In this way, our analysis is a better replication of the original FINDRISC analysis that also utilised logistic regression. Furthermore, other potentially important and widely available risk factors (e.g., sex, smoking) were not considered that could have improved our prediction model.

### 4.4 Conclusion

Our study represents a crucial step given that no previous validation was performed in a prospective population‐based cohort in Hungary. We found that the reweighting of the individual risk factors of the original score only moderately improves discrimination and reclassification, thus suggesting that recalibration (resetting the cutoff value based on the underlying diabetes risk in each population) could be sufficient before its wide scale use. Furthermore, we found substantially worse overall discrimination in our cohort compared with the derivation cohort, suggesting that population characteristics (especially age distribution) could have a huge effect on model performance. Our results suggest that diabetes risk prediction could worsen in older populations, limiting its usability.

## Author Contributions

Concept and study design: A.G.T., P.S., Z.S., B.M. and Z.B., methodology: A.G.T., I.P., B.A.D., P.S., Z.S., Z.B. and L.Z.K., validation: A.G.T. and Z.B., resources: A.G.T., B.M., P.S., Z.S. and Z.B., writing – original draft preparation: Z.B., I.P., B.A.D. and L.Z.K., writing – review and editing: A.G.T. and B.M., visualization: A.G.T. and L.Z.K., supervision: A.G.T, B.M. and Z.B., project administration: Z.B., funding acquisition: A.G.T., B.M., P.S., Z.S. and Z.B. All authors have read and agreed to the published version of the manuscript.

## Funding

This study was supported by the National Research, Development, and Innovation Fund (NRDIF) of Hungary (Grants NVKP_16‐1–2016–0017 and TKP2021‐NVA‐15) and by the European Union (Grant RRF‐2.3.1–21‐2022–00003). AGT was supported by the UK Medical Research Council (S011676) and the (TKP2021‐NKTA‐47) grant provided by the Ministry of Innovation and Technology of Hungary from the NRDIF, financed under the 2021 Thematic Excellence Programme funding scheme. A.G.T., Z.B. and L.Z.K. were supported by the (2020‐2.1.1‐ED‐2023‐00255) grant provided by the Ministry of Culture and Innovation of Hungary from the NRDIF, financed under the 2020 Government Guarantee Fund funding scheme for the execution of the HORIZON‐HLTH‐2023‐TOOL‐05 (Project ID: 101136305) programme.

## Ethics Statement

The study was approved by the Medical Research Council of Hungary and conformed to the Declaration of Helsinki.

## Consent

Written informed consent was obtained from all participants.

## Conflicts of Interest

The authors declare no conflicts of interest.

## Supporting information


Supporting Information S1

**Figure S1**: Study flowchart.

## Data Availability

The data that support the findings of this study are available on request from the corresponding author. The data are not publicly available due to privacy or ethical restrictions.
