# Supplementary material for: Validation of the Finnish Diabetes Risk Score (FINDRISC) in a Central‐European Population for the Prediction of Cumulative Incidence of Type 2 Diabetes Over 8‐Years—Follow‐Up of the Budakalász Health Examination Survey (BHES)
Source: Diabetes Metab Res Rev. 2025 Nov 12;41(8):e70105. doi: 10.1002/dmrr.70105 (PMC12611636; doi:10.1002/dmrr.70105)
Supplement: Supplementary file 1 — Supporting Information S1 [file DMRR-41-e70105-s001.docx]

**Supplementary Appendix**

**Validation of the Finnish Diabetes Risk Score (FINDRISC) in a Central-European population for the prediction of cumulative incidence of type 2 diabetes over 8-years – Follow-up of the Budakalász Health Examination Survey (BHES)**

Authors: Zsolt Bagyura^1^, Loretta Zsuzsa Kiss^1*^, István Panykó^2,3,4^, Beatrix Annamária Domján^2, 3^, Pál Soós^1^, Zsolt Szelid^1^, Béla Merkely^1^, Ádám Gyula Tabák^2,5,6^

1 Heart and Vascular Center, Semmelweis University, Városmajor utca 68., Budapest H-1122, Hungary

2 Department of Internal Medicine and Oncology, Semmelweis University Faculty of Medicine, Korányi Sándor u. 2/a., H-1083 Budapest, Hungary

3 Károly Rácz Conservative Medicine Division, Doctoral College, Semmelweis University, Üllői út 26., H-1085 Budapest, Hungary

4 Department of Gynaecology and Obstetrics, Komárom-Esztergom County Szent Borbála Hospital, Dózsa György út 77., H-2800 Tatabánya, Hungary

5 Institute of Preventive Medicine and Public Health, Semmelweis University Faculty of Medicine, Nagyvárad tér 4., H-1089 Budapest, Hungary

6 UCL Brain Sciences, University College London, 149 Tottenham Court Road, W1T 7NF London, United Kingdom

**Supplementary Methods**

***Calculation of FINDRISC according to the weighting published by Lindström et al.^17^***

Age: 0 point - <45 years, 2 points – 45-54 years, 3 points – 55-64 years, and 4 points >64 years; BMI: 0 point - < 25, 1 point – 25-30, and 3 points >30 kg/m^2^; waist: 0 point - <94/<80 (men/women), 3 points – 94-102/80-88, and 4 points - >102/>88 cm; at least 30 minutes of physical activity: 0 point – yes, 2 points – no; daily fruit and vegetable consumption: 0 point – yes, 1 point – no; treated hypertension in the medical history: 0 point – no, 2 points – yes; high blood glucose in the medical history: 0 point – no, 5 points – yes; and family history of diabetes mellitus: 0 point – no, 3 point – yes but no first degree relative with diabetes, and 5 points – first degree relative with diabetes mellitus.

Translation of the beta coefficients to points: for beta = 0.01–0.2, the score is 1; for beta = 0.21–0.8, the score is 2; for beta = 0.81–1.2, the score is 3; for beta = 1.21–2.2, the score is 4; and for beta >2.2, the score is 5 where the category with the lowest hypothetical risk has a value of 0.

1. Lindström J, Tuomilehto J. The diabetes risk score: a practical tool to predict type 2 diabetes risk. *Diabetes Care.* 2003;26(3):725-731.
