# Supplementary figures and images for: Validation of the Finnish Diabetes Risk Score (FINDRISC) in a Central‐European Population for the Prediction of Cumulative Incidence of Type 2 Diabetes Over 8‐Years—Follow‐Up of the Budakalász Health Examination Survey (BHES)
Source: Diabetes Metab Res Rev. 2025 Nov 12;41(8):e70105. doi: 10.1002/dmrr.70105 (PMC12611636; doi:10.1002/dmrr.70105)

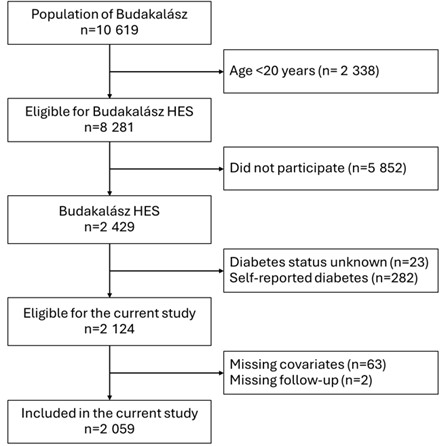

Supplement: Supplementary file 2 — Figure S1: Study flowchart. [file DMRR-41-e70105-s002.jpg]
